# Clonal hematopoiesis of indeterminate potential is associated with acute kidney injury

**DOI:** 10.1038/s41591-024-02854-6

**Published:** 2024-03-07

**Authors:** Caitlyn Vlasschaert, Cassianne Robinson-Cohen, Jianchun Chen, Elvis Akwo, Alyssa C. Parker, Samuel A. Silver, Pavan K. Bhatraju, Hannah Poisner, Shirong Cao, Ming Jiang, Yinqiu Wang, Aolei Niu, Edward Siew, Joseph C. Van Amburg, Holly J. Kramer, Anna Kottgen, Nora Franceschini, Bruce M. Psaty, Russell P. Tracy, Alvaro Alonso, Dan E. Arking, Josef Coresh, Christie M. Ballantyne, Eric Boerwinkle, Morgan Grams, Ming-Zhi Zhang, Bryan Kestenbaum, Matthew B. Lanktree, Michael J. Rauh, Raymond C. Harris, Alexander G. Bick

**Affiliations:** 1 Department of Medicine, Queen’s University, Kingston, Ontario Canada; 2 Division of Nephrology and Hypertension, Department of Medicine, Vanderbilt O’Brien Center for Kidney Disease, Vanderbilt University Medical Center, Nashville, TN USA; 3 Division of Genetic Medicine, Department of Medicine, School of Medicine, Vanderbilt University, Nashville, TN USA; 4 Division of Pulmonary, Critical Care and Sleep Medicine, Department of Medicine, University of Washington, Seattle, WA USA; 5 Departments of Public Health Sciences and Medicine, Loyola University Chicago, Maywood IL, USA; 6 Institute of Genetic Epidemiology, Faculty of Medicine and Medical Center, University of Freiburg, Freiburg, Germany; 7 Department of Epidemiology, Bloomberg School of Public Health, Johns Hopkins University, Baltimore, MD USA; 8 Department of Epidemiology, Gillings School of Global Public Health, University of North Carolina, Chapel Hill, NC USA; 9 Cardiovascular Health Research Unit, Departments of Medicine, Epidemiology and Health Systems and Population Health, University of Washington, Seattle, WA USA; 10 Kaiser Permanente Washington Health Research Institute, Seattle, WA USA; 11 Pathology and Biochemistry, University of Vermont, Burlington, VT USA; 12 Department of Epidemiology, Rollins School of Public Health, Emory University, Atlanta, GA USA; 13 McKusick-Nathans Institute, Department of Genetic Medicine, John Hopkins University School of Medicine, Baltimore, MD USA; 14 Welch Center for Prevention, Epidemiology, and Clinical Research, Johns Hopkins University, Baltimore, MD USA; 15 Department of Medicine, Baylor College of Medicine, Houston, TX USA; 16 Human Genetics Center, The University of Texas Health Science Center at Houston, Houston, TX USA; 17 Division of Nephrology, Department of Internal Medicine, Johns Hopkins University, Baltimore, MD USA; 18 Kidney Research Institute, Division of Nephrology, Department of Medicine, University of Washington, Seattle, WA USA; 19 Department of Medicine and Department of Health Research Methods, Evidence and Impact, McMaster University, Hamilton, Ontario Canada; 20 St. Joseph’s Healthcare Hamilton, Hamilton, Ontario Canada; 21 Population Health Research Institute, Hamilton, Ontario Canada; 22 Department of Pathology and Molecular Medicine, Queen’s University, Kingston, Ontario Canada; 23 U.S Department of Veterans Affairs, Nashville, TN USA

**Keywords:** Genetics, Acute kidney injury, Haematological diseases

## Abstract

Age is a predominant risk factor for acute kidney injury (AKI), yet the biological mechanisms underlying this risk are largely unknown. Clonal hematopoiesis of indeterminate potential (CHIP) confers increased risk for several chronic diseases associated with aging. Here we sought to test whether CHIP increases the risk of AKI. In three population-based epidemiology cohorts, we found that CHIP was associated with a greater risk of incident AKI, which was more pronounced in patients with AKI requiring dialysis and in individuals with somatic mutations in genes other than *DNMT3A*, including mutations in *TET2* and *JAK2*. Mendelian randomization analyses supported a causal role for CHIP in promoting AKI. Non-*DNMT3A*-CHIP was also associated with a nonresolving pattern of injury in patients with AKI. To gain mechanistic insight, we evaluated the role of *Tet2*-CHIP and *Jak2*^*V617F*^-CHIP in two mouse models of AKI. In both models, CHIP was associated with more severe AKI, greater renal proinflammatory macrophage infiltration and greater post-AKI kidney fibrosis. In summary, this work establishes CHIP as a genetic mechanism conferring impaired kidney function recovery after AKI via an aberrant inflammatory response mediated by renal macrophages.

## Abstract (web summary)

Clonal hematopoiesis, which increases with age and is implicated in a variety of age-related diseases, is shown here to be associated with a greater risk of acute kidney injury and worse outcome following injury, as demonstrated using multiple patient cohorts, Mendelian randomization analysis and mechanistic studies in mouse disease models.

## Main

Acute kidney injury (AKI) affects more than 1 in 5 hospitalized adults worldwide^1,2^ and is associated with substantial healthcare costs and patient mortality, greater than that of heart failure or diabetes^3^. AKI is characterized by an inflammatory and fibrotic response to an initial insult, most commonly kidney hypoperfusion, leading to a quantifiable impairment in kidney function based on serum markers and urine output^4^. After AKI, there is a remarkable heterogeneity of outcomes. Only about half of cases with AKI return to baseline kidney function within 3 months, while many have residual kidney damage^5^. Recognized patient factors that predispose to AKI and to progression from AKI to chronic kidney disease largely consist of nonmodifiable clinical risk factors such as age^2^. To date, there have been no identified genetic factors that predispose to AKI or AKI outcomes.

Dysregulated inflammatory responses in macrophages and other inflammatory cells can occur in the setting of clonal hematopoiesis of indeterminate potential (CHIP), a common age-related hematological process characterized by the clonal expansion of hematopoietic stem cells (HSCs) and their progeny after an acquired genetic mutation (commonly in *DNMT3A*, *TET2*, *ASXL1* and *JAK2*). While less than 0.5% of cases with CHIP per year progress to overt hematological cancer^6,7^, CHIP is associated with an estimated 40% greater risk of mortality^8^ largely because of disease beyond the hematopoietic system, including cardiovascular disease^9–12^, pulmonary disease^13,14^, liver disease^15^ and other inflammatory conditions^16–19^. CHIP may influence kidney health: it has been associated with poorer cross-sectional kidney function and kidney function decline in the general population, and chronic kidney disease (CKD) progression^20–22^. Experimental recapitulation of CHIP by transplanting a small fraction of HSCs with pathogenic *Tet2* mutations in mice showed that *Tet2*-deficient cells readily replace resident macrophage populations in the kidney and liver^15,23^, and myeloid cells have pivotal roles in response to injury, repair and management of the kidney microenvironment^24–26^.

In this study, we tested the hypothesis that CHIP is a risk factor for AKI. We first show that CHIP is associated with incident AKI in three large population-based cohorts and is more pronounced for CHIP with driver mutations in genes other than *DNMT3A*, such as *TET2* and *JAK2*. We then show that non-*DNMT3A*-CHIP is associated with a nonresolving AKI pattern in the Assessment, Serial Evaluation, and Subsequent Sequelae in Acute Kidney Injury (ASSESS)-AKI and Vanderbilt’s Biobank (BioVU) cohorts. Finally, we show that AKI severity is more pronounced and AKI recovery is impaired in mouse models with *Tet2*-CHIP and *Jak2*^*V617F*^-CHIP across both ischemia–reperfusion injury (IRI) and unilateral ureteral obstruction (UUO) AKI models, with risk mediated by an aberrant inflammatory response in CHIP-mutant renal macrophages.

## Results

### CHIP and incident AKI

**Extended Data Fig. 1:**
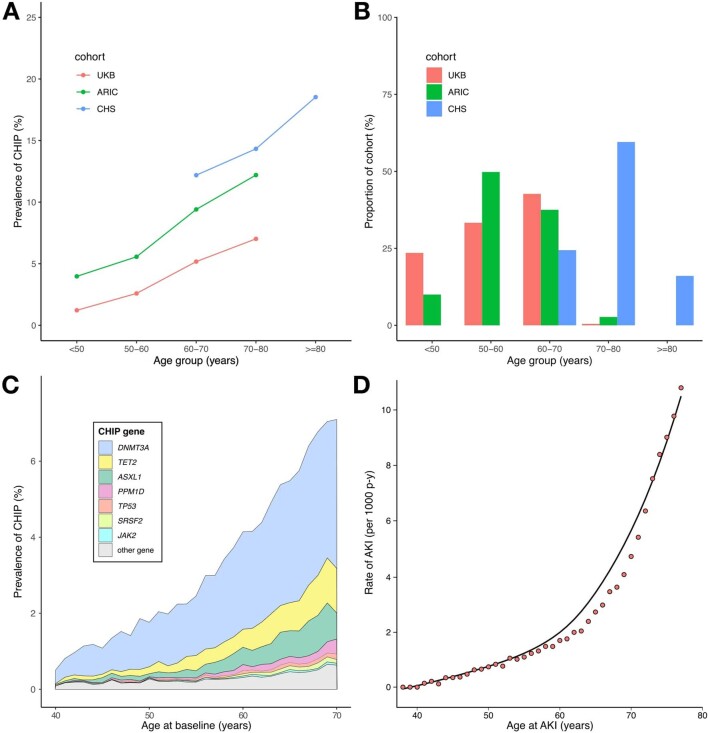
Distribution of CHIP mutations and AKI rates by age. **A)** Distribution of CHIP prevalence by age group across the cohorts included in the study. **B)** Proportion of individuals in each age group across the cohorts. **C)** Prevalence of major CHIP gene subtypes by age at baseline in the UKB. **D)** Rate of AKI across ages observed during follow-up.

We first assessed the association between CHIP and incident AKI in the UK Biobank (UKB). The mean baseline age was 57 ± 8 (s.d.) years, the mean baseline estimated glomerular filtration rate (eGFR) was 95 ± 14 ml per min per 1.73 m^2^ (Supplementary Table 1 and Extended Data Fig. 1a). The prevalence of CHIP was 3.4% and increased with age (Extended Data Fig. 1b). *DNMT3A* was the most commonly mutated gene followed by *TET2* and *ASXL1*. There were 15,736 incident AKI events among 428,793 participants (3.1 events per 1,000 person-years); the prevalence of AKI increased with age (Extended Data Fig. 1c).

**Fig. 1:**
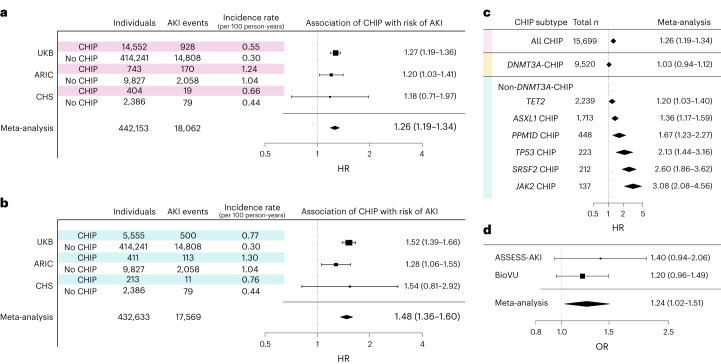
CHIP is associated with a greater risk of incident AKI in three population-based cohorts. **a**, Random effects meta-analysis of the association between CHIP and risk of incident AKI in the UKB, ARIC and CHS cohorts. **b**, Random effects meta-analysis of the association between CHIP driven by mutations in genes other than *DNMT3A* (non-*DNMT3A*-CHIP) and risk of incident AKI in the UKB, ARIC and CHS cohorts. **c**, Random effects meta-analysis of the risk of incident AKI across major CHIP driver genes. For the analyses in **a**–**c**, Cox proportional hazards analyses were conducted, adjusting for age, age^2^, sex, baseline eGFR, baseline smoking status, diabetes and hypertension, as well as either ten principal components of genetic ancestry (UKB) or self-reported ethnicity (ARIC and CHS). These results are presented as HRs with 95% CIs. **d**, MR examining the association between CHIP and AKI risk. The results shown represent a random effects meta-analysis of conventional multiplicative random effects inverse variance weighted (IVW) estimators. These results are presented as odds ratios (ORs) with 95% CIs.

**Extended Data Fig. 2:**
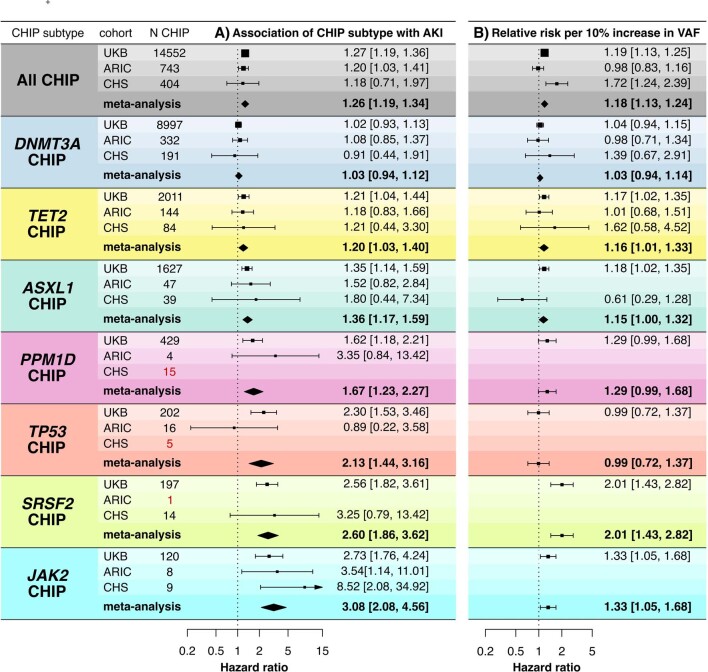
Associations of individual CHIP genes with AKI risk in three prospective cohorts. **A)** Risk of AKI by binary CHIP status. **B)** Risk of AKI among CHIP carriers per 10% increase in variant allele fraction (VAF). Analyses that did not converge due to small sample size or lack of events in the CHIP subgroup are left as blank rows. All analyses were adjusted for age, age^2^, sex, baseline eGFR, baseline smoking status, diabetes, and hypertension, as well as either 10 PCs of genetic ancestry (UKB) or self-reported race/ethnicity (ARIC & CHS).

**Extended Data Fig. 3:**
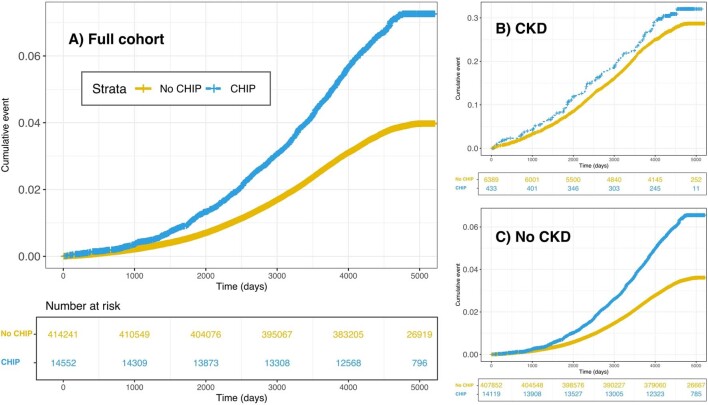
Kaplan-Meier plots illustrating cumulative AKI events as a function of time in the UKB cohort. Cumulative AKI events are shown for (**A**) the whole cohort, (**B**) those with baseline CKD and (**C**) those without baseline CKD.

CHIP was associated with a 34% greater risk of incident AKI (hazard ratio (HR) = 1.34, 95% confidence interval (CI) = 1.29–1.40, *P* < 0.0001) in a Cox proportional hazards model in fully adjusted analyses (Fig. 1a). The association between CHIP and AKI was stronger when AKI was limited to cases receiving dialysis (AKI-D) (HR = 1.65, 95% CI = 1.24–2.20, *P* = 0.001). The risk for AKI associated with CHIP was enhanced in individuals with mutations in genes other than *DNMT3A* (Fig. 1b; HR = 1.54, 95% CI = 1.41–1.68 for any AKI and HR = 2.18, 95% CI = 1.51–3.15 for AKI-D, *P* < 0.0001). Evaluating this association at the individual driver gene level identified significant associations across the most common genes other than *DNMT3A* (Fig. 1c): *TET2*-CHIP (HR = 1.21, 95% CI = 1.04–1.44); *ASXL1*-CHIP (HR = 1.35, 95% CI = 1.14–1.59); *PPM1D*-CHIP (HR = 1.62, 95% CI = 1.18–2.21); *TP53*-CHIP (HR = 2.30, 95% CI = 1.53–3.46); *SRSF2*-CHIP (HR = 2.56, 95% CI = 1.82–3.61); and *JAK2*-CHIP (HR = 2.73, 95% CI = 1.76–4.24). Conversely, *DNMT3A*-CHIP was not significantly associated with AKI (HR = 1.02, 95% CI = 0.93–1.13). Additionally, the risk of AKI was proportional to the variant allele fraction (VAF) among CHIP carriers (HR = 1.19, 95% CI = 1.13–1.25 per 10% increase in VAF; Extended Data Fig. 2b), suggesting a dose effect. Although the absolute risk for AKI was higher among those with baseline CKD, CHIP conferred a similar absolute risk difference among those with and those without baseline CKD (Extended Data Fig. 3).

Next, we assessed the association of CHIP with incident AKI in two prospective cohort studies: the Atherosclerosis Risk in Communities (ARIC) cohort and the Cardiovascular Health Study (CHS)^27,28^. The mean baseline age was 57 ± 4 (s.d.) years in the ARIC cohort and 72 ± 5 years in the CHS cohort. Mean baseline eGFR was 96 ± 15 ml per min per 1.73m^2^ in ARIC and 68 ± 16 ml per min per 1.73 m^2^ in CHS. As reported previously, the prevalence of CHIP was 7.6% in ARIC and 14.5% in CHS^29^ (age distribution is shown in Extended Data Fig. 1a). The baseline characteristics for these cohorts are listed in Supplementary Table 1.

There were 2,228 events among 10,570 individuals in ARIC (10.5 events per 1000 person-years) and 98 events among 2,790 individuals in CHS (4.7 events per 1000 person-years). The median follow-up times were 9 years (interquartile range (IQR) = 8–22) in ARIC and 7 years (IQR = 4–7) in CHS. CHIP was associated with a 20% greater risk of AKI in a meta-analysis of these two cohorts (HR = 1.20; 95% CI = 1.03–1.39), leading to an overall risk of 26% after meta-analysis with the UKB (HR = 1.26, 95% CI = 1.19–1.34; Fig. 1a). Consistent with what was observed in the UKB, the point estimate of the magnitude of relative risk for AKI was higher for individuals with non-*DNMT3A*-CHIP (Fig. 1b; meta-analyzed HR = 1.28, 95% CI = 1.06–1.55) and across many of the top individual genes in this subgroup (Extended Data Fig. 2), while *DNMT3A*-CHIP was not associated with AKI. As seen in the UKB, the absolute risk of AKI was higher among those with CKD, but the risk difference was not different according to CHIP status between the CKD and non-CKD subgroups.

Finally, we performed two-sample Mendelian randomization (MR) analyses to investigate whether a causal role for CHIP in AKI could be inferred. As shown in Fig. 1d, genetically predicted CHIP risk was significantly associated with greater odds of AKI with a meta-analysis MR point estimate similar to the findings from the observational data. The findings were consistent across statistical models (Supplementary Table 2). These data provide another layer of evidence in support of a causal role for CHIP in the pathogenesis of AKI.

### CHIP and recovery from AKI

**Fig. 2:**
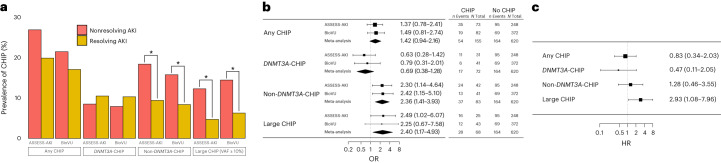
CHIP is associated with impaired recovery from AKI in the ASSESS-AKI and BioVU cohorts. **a**, Prevalence of CHIP among individuals with a resolving AKI pattern in ASSESS-AKI (*n* = 191) and BioVU (*n* = 88) compared to a nonresolving AKI pattern (*n* = 130 for ASSESS-AKI and *n* = 366 for BioVU), as defined by Bhatraju et al.^5^. **b**, Odds of nonresolving AKI pattern according to CHIP status, adjusted for age, sex, baseline creatinine, AKI stage, smoking status, ethnicity and history of diabetes, hypertension and cardiovascular disease. **c**, Risk of significant kidney function impairment (primary study composite outcome of ESKD or eGFR decline by ≥50%) over 5 years of follow-up among ASSESS-AKI participants with baseline AKI according to CHIP status, adjusted for age, sex, baseline creatinine, AKI stage, smoking status, ethnicity and history of diabetes, hypertension and cardiovascular disease. *P < 0.05, ***P < 0.001.

We assessed whether CHIP was associated with patterns of AKI recovery in the ASSESS-AKI cohort, which enrolled 769 individuals with AKI events during a hospitalization and longitudinally tracked their clinical outcomes over 5 years^30^. Among 321 individuals with AKI and DNA samples available for CHIP ascertainment, 74 (23%) had CHIP. Among individuals with AKI, non-*DNMT3A*-CHIP and large CHIP clones (VAF ≥ 10%) were more than twice as common among individuals with a nonresolving AKI pattern compared to those with resolving AKI (Fig. 2a), including after adjusting for age and other relevant covariates (Fig. 2b). Additionally, large CHIP clones were associated with an increased risk of the study primary outcome (incident kidney failure or 50% decline in eGFR over 5 years; HR = 2.9, 95% CI = 1.1–8.0; Fig. 2c). We assessed whether these results could be replicated in an independent cohort. We CHIP-genotyped 454 individuals who had a noted AKI event and two or more subsequent creatinine measurements to assess AKI recovery patterns in the BioVU biobank. Again we identified that non-DNMT3A-CHIP and large CHIP were associated with a nonresolving AKI pattern, including in adjusted analyses (Fig. 2a,b). In a meta-analysis of both cohorts, non-*DNMT3A*-CHIP and large CHIP were associated with a 2.36-fold (95% CI = 1.41–3.93) and 2.40-fold (95% CI = 1.17–4.93) higher odds of nonresolving AKI, respectively (Fig. 2b), whereas *DNMT3A*-CHIP was not associated with AKI recovery patterns (HR = 0.69, 95% CI = 0.38–1.28).

### CHIP and kidney injury severity in mouse models

**Extended Data Fig. 4:**
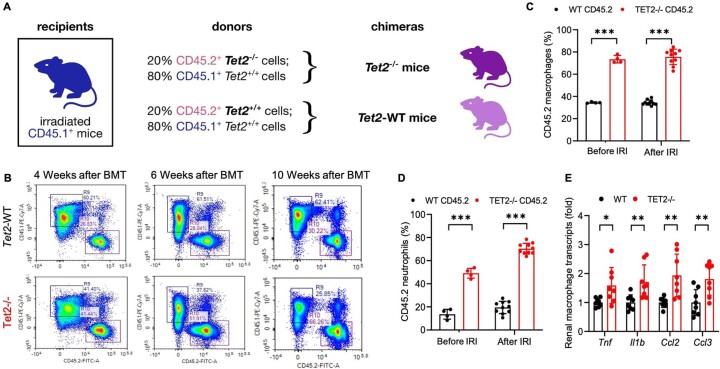
Creation of *Tet2*^*-/–*^CHIP mice and WT controls. **A)** Schema for production of wild type and *Tet2*^*-/-*^ mice. **B)** Representative images of flow cytometry of clonal hematopoietic expansion in *Tet2*^*-/-*^ mice. **C&D)** *Tet2*^*-/-*^ macrophages and neutrophils in kidneys without injury (n = 4 mice) and 8 days after ischemic injury (n = 10 mice). **E)** Kidney macrophage mRNA of proinflammatory cytokines *Tnf, Il1b, Ccl2* and *Ccl3* in *Tet2*^*-/-*^ and WT mice without injury (n = 8 mice). Data were analyzed using 2-tailed Student’s t-test or two-way ANOVA followed by Tukey’s or Bonferroni’s post hoc tests and presented as mean ± SEM. * p < 0.05, ** p < 0.01, *** p < 0.001, scale bar = 50 µm.

We sought to leverage CHIP mouse models to obtain mechanistic insights into how CHIP contributed to kidney injury. Because non-*DNMT3A*-CHIP was most strongly associated with AKI outcomes in our epidemiological studies, we leveraged a well-established mouse model of *TET2*-CHIP^9,31^, the most common type of non-*DNMT3A*-CHIP. Briefly, we performed a bone marrow transplant containing 20% CD45.2^+^*Tet2*^−/−^ cells and 80% CD45.1^+^*Tet2*^+/+^ cells in lethally irradiated mice (Extended Data Fig. 4a). Control mice received a bone marrow transplant of 20% CD45.2^+^*Tet2*^+/+^ cells and 80% CD45.1^+^*Tet2*^+/+^ cells. These mice are referred to as *Tet2*^−/−^ and wild-type (WT), respectively, throughout the text. When studied 10 weeks after bone marrow transplantation, mice receiving the *Tet2* WT CD45.2 cells had a minority of both kidney macrophage and neutrophil CD45.2 cells in control kidneys. In contrast, the *Tet2*^−/−^ mice had a significant increase of *Tet2*^−/−^ cells in the intrinsic myeloid kidney cell population (Extended Data Fig. 4c,d).

**Fig. 3:**
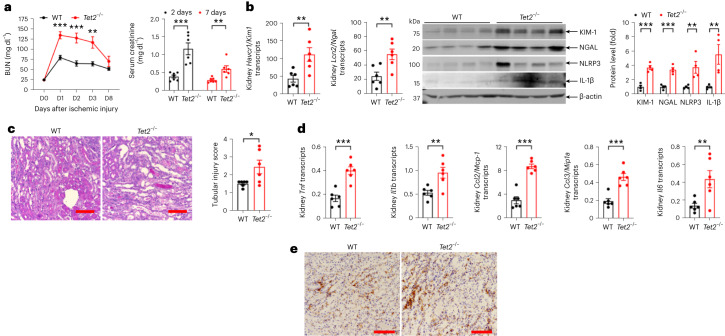
Early response of hematopoietic deletion of *Tet2* to ischemic kidney injury in mice. **a**, BUN and serum creatinine in WT and *Tet2*^−/−^ mice subjected to ischemic kidney injury (*n* = 6 mice each). **b**, mRNA of the tubule injury markers *Kim-1* and *Ngal* (*n* = 6 mice each) and quantification of immunoreactive protein kidney levels of the kidney injury markers *Kim-1* and *Ngal*, as well as the inflammatory markers *Nlrp3* and *Il-1β* in the kidneys of WT and *Tet2*^−/−^ mice 8 days after injury (*n* = 4 mice each). **c**, Representative image of kidney histology with Periodic Acid–Schiff stain (left) and quantitation of the tubule injury score (*n* = 6 mice each, right). **d**, Kidney mRNA expression of the proinflammatory cytokines *Tnf*, *Il1b*, *Ccl2*, *Ccl3* and *Il6* in *Tet2*^−/−^ and WT mice (*n* = 6 mice each). **e**, Representative image of macrophage infiltration (F4/80 immunostaining). Data were analyzed using a two-tailed Student’s *t*-test or two-way analysis of variance (ANOVA) followed by Tukey’s or Bonferroni’s post hoc tests and presented as the mean ± s.e.m. *P < 0.05, **P < 0.01, ***P < 0.001. Scale bar, 50 µm. Source data

**Extended Data Fig. 5:**
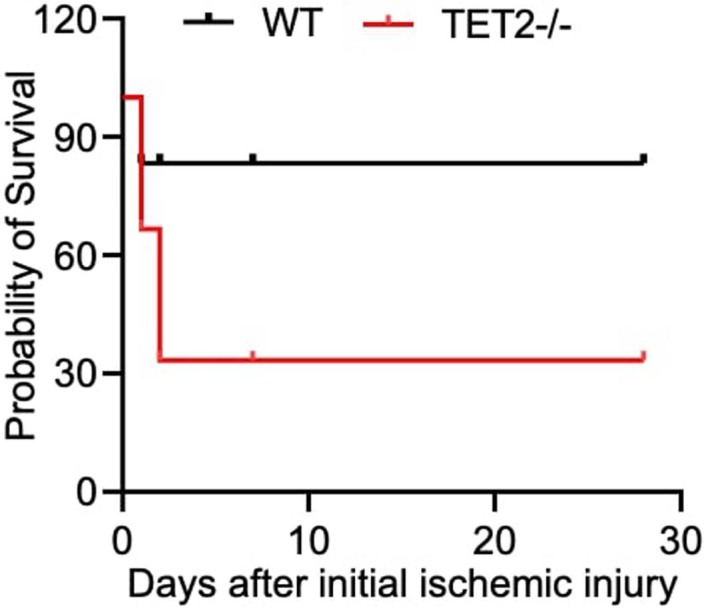
Survival curve of wild type and *Tet2*^*-/-*^ mice following severe kidney injury. Survival curve of WT and *Tet2*^*-/-*^ mice following 33.5 minutes of renal pedicle clamping (n = 10 mice each).

*Tet2*^−/−^ mice had an exaggerated kidney injury pattern after IRI. Blood urea nitrogen (BUN) increased within 24 h from 25 mg dl^−1^ to approximately 75 mg dl^−1^ in WT mice and decreased over the subsequent 8 days, whereas the BUN increases after the same ischemic insult in *Tet2*^−/−^ mice were significantly higher (*P* < 0.01; Fig. 3a). Similarly, serum creatinine was also significantly higher in *Tet2*^−/−^ mice than WT mice at both 2 and 7 days after ischemic injury. When mice were subjected to more extensive ischemic injury, there was increased early mortality in *Tet2*^−/−^ mice (4 of 6) compared to WT mice (1 of 7; Extended Data Fig. 5), which is consistent with increased sensitivity to ischemic injury.

*Tet2*^−/−^ mice had an exaggerated histological and molecular kidney injury pattern after IRI. Eight days after ischemic injury, the kidneys of *Tet2*^−/−^ mice had increased expression of mRNA and protein for the tubule injury markers KIM-1 and NGAL^32,33^ (Fig. 3b); histological analysis indicated significantly more tubule injury (Fig. 3c). The kidneys of *Tet2*^−/−^ mice had increased mRNA for the proinflammatory cytokines *Tnf*, *Il6* and *Il1b*, and the chemokines *Ccl2* and *Ccl3* (Fig. 3d), as well as increased immunostaining for the macrophage marker F4/80 (Fig. 3e).

**Fig. 4:**
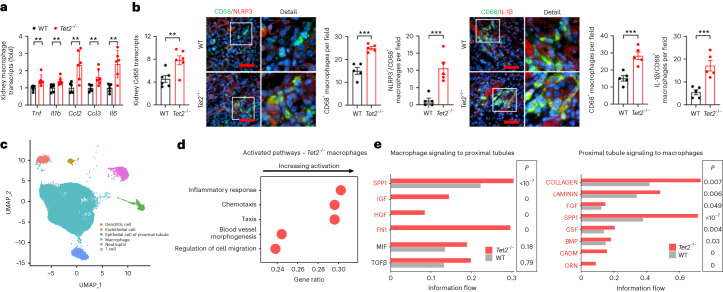
*Tet2*-deficient macrophages are hyperinflammatory in early ischemic kidney injury. **a**, Kidney macrophage mRNA expression of the proinflammatory cytokines and chemokines *Tnf*, *Il1b*, *Ccl2*, *Ccl3* and *Il6* 8 days after ischemic injury in *Tet2*^−/−^ and WT mice (*n* = 6 mice each). **b**, Kidney *Cd68* mRNA expression in *Tet2*^−/−^ and WT mice (*n* = 6 mice each) and representative images and quantification of immunoreactive CD68 and its colocalization with immunoreactive NLRP3 and IL-β (*n* = 5 mice each). **c**, Representative uniform manifold approximation and projection (UMAP) plot of cell types examined in the scRNA-seq analyses of *Tet2*^−/−^ and WT mouse kidney cell samples enriched for CD45^+^ (hematopoietic) cells. **d**, Gene set enrichment analysis (GSEA) analysis of WT and *Tet2*^−/−^ macrophages after scRNA-seq. **e**, CellChat analyses of cell–cell communication pathways between macrophages and proximal tubules epithelial cells isolated from *Tet2*^−/−^ and WT mouse kidneys. Data were analyzed using a two-tailed Student’s *t*-test or two-way ANOVA followed by Tukey’s or Bonferroni’s post hoc tests and are presented as the mean ± s.e.m. **P < 0.01, ***P < 0.001; scale bar, 50 µm.

**Fig. 5:**
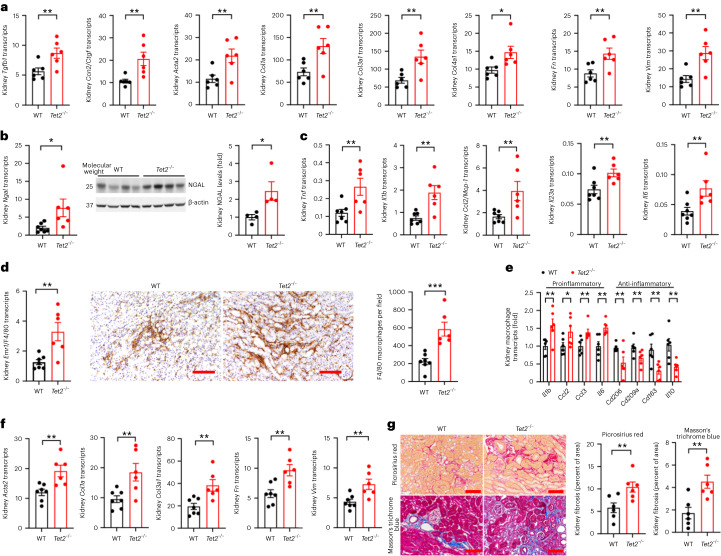
Increased kidney interstitial fibrosis after ischemic kidney injury with hematopoietic deletion of *Tet2*. **a**, Kidney mRNA of the profibrotic markers *Tgfb1*, *Ccn2*, *Acta2*, *Col1a*, *Col3a1*, *Col4a1*, *Fn* and *Vim* 8 days after injury in *Tet2*^−/−^ and WT mice (*n* = 6 mice each). **b**, Kidney *Ngal1* mRNA (*n* = 6 mice each) and protein expression (*n* = 4 mice each) 28 days after injury in *Tet2*^−/−^ and WT mice. **c**, Kidney mRNA for the inflammatory cytokines *Tnf*, *Il1b*, *Ccl2*, *Il23a* and *Il6* in kidneys 28 days after injury in *Tet2*^−/−^ and WT mice (*n* = 6 mice each). **d**, Kidney *Emr1*/F480 mRNA quantification and representative F4/80 macrophage immunohistochemistry (IHC) 28 days after injury in *Tet2*^−/−^ and WT mice (*n* = 6 mice each). **e**, Kidney macrophage mRNA expression of the proinflammatory cytokines *Il1b*, *Ccl2*, *Ccl3* and *Il6* and anti-inflammatory, pro-reparative cytokines *Cd206*, *Cd290a*, *Cd163* and *Il10* in kidney macrophages isolated from WT and *Tet2*^−/−^ mice 28 days after injury (*n* = 6 mice each). **f**, Kidney mRNA for the fibrotic markers *Acta2*, *Col1a*, *Col3a1*, *Fn* and *Vim* 28 days after injury in *Tet2*^−/−^ and WT mice (*n* = 6 mice each). **g**, Representative images of kidney histology stained with Masson trichrome blue and Picrosirius red, and quantification of interstitial collagen expression in the kidneys of WT and *Tet2*^−/−^ mice 28 days after injury (*n* = 6 mice each). Data were analyzed using a two-tailed Student’s *t*-test or two-way ANOVA followed by Tukey’s or Bonferroni’s post hoc tests and are presented as the mean ± s.e.m. *P < 0.05, **P < 0.01, ***P < 0.001. Scale bar, 50 µm. Source data

**Extended Data Fig. 6:**
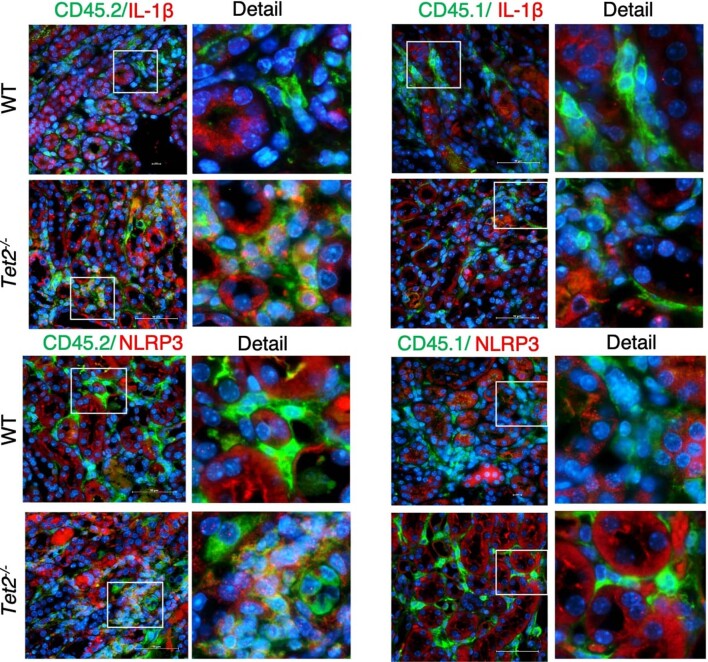
Increased colocalization of *Tet2*^*-/-*^ CD45.2 cells but not *Tet2*^*+/+*^ CD45.2 cells nor *Tet2*^*+/+*^ CD45.1 cells with immunoreactive NLRP3 and IL-β. Representative of n = 4 colocalization studies of *Tet2*^*-/-*^ CD45.2 cells or *Tet2*^*+/+*^ CD45.2 cells and *Tet2*^*+/+*^ CD45.1 cells with immunoreactive NLRP3 and IL-β. scale bar = 50 µm.

*Tet2*^−/−^ pathology was localized to the *Tet2*^−/−^ mutant renal macrophages. Macrophages isolated from the kidneys of *Tet2*^−/−^ mice had increased mRNA expression of the proinflammatory cytokines *Tnf*, *Il6* and *Il1b* both at baseline (Extended Data Fig. 4e) and after ischemic injury (Fig. 4a). Compared to WT mice, *Tet2*^−/−^ mice also had increased kidney mRNA and immunoreactive expression of the macrophage inflammatory marker CD68 (Fig. 4b) and expressed higher levels of the NLRP3 inflammasome and its product interleukin-1β (IL-1β) (Fig. 3b), both of which colocalized with CD68^+^ macrophages (Fig. 4b). Importantly, the increased coexpression was only seen in the CD45.2 (*Tet2*^−/−^) cells of *Tet2*^−/−^ mice and not in the CD45.1 (*Tet2*^+/+^) cells of *Tet2*^*−/−*^ mice, nor in either cell type in WT mice (Extended Data Fig. 6). Single-cell RNA sequencing (scRNA-seq) analyses of *Tet2*^−/−^ and WT mouse kidney cell samples enriched for CD45^+^ (hematopoietic) cells revealed broad upregulation of inflammatory and chemotaxis pathways in *Tet2*^−/−^ macrophages (Fig. 4c,d). Using CellChat, a computational tool that enables inferences about cell–cell communication in scRNA-seq datasets, we identified increased inflammatory and fibrotic signaling between macrophages and proximal tubular epithelial cells—the main cellular target of ischemic kidney injury—in *Tet2*^−/−^ mice compared to WT mice (Fig. 4e). Targeted measurement of profibrotic (*Tgfb*, *Ctgf*, *Acta2*) and extracellular matrix-associated (*Col1a1*, *Col3a1*, *Fn* and *Vim*) gene expression using quantitative PCR (qPCR) congruently showed increased mRNA levels of these genes in the kidneys of *Tet2*^−/−^ mice compared to WT mice 8 days after ischemic injury (Fig. 5a).

**Extended Data Fig. 7:**
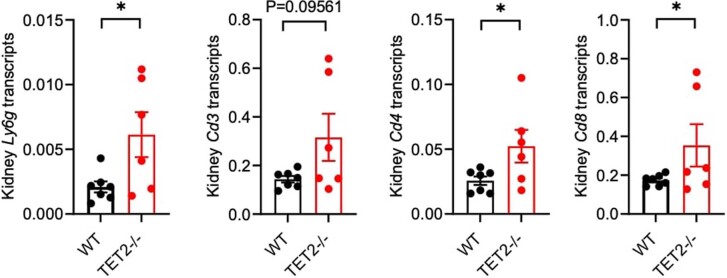
28 days after ischemic injury, kidneys of *Tet2*^*-/-*^ mice had increased markers for neutrophils, total T cells, and CD4 and CD8 T cells. mRNA markers for neutrophils (*Ly6g*), total T cells (*Cd3*) and CD4 and CD8 T cells in kidneys of WT and *Tet2*^*-/-*^ mice 28 days after ischemic injury (n = 7 WT mice and n = 6 *Tet2*^*-/-*^ mice). Data were analyzed using 2 tailed Student’s t-tests and presented as mean ± SEM. *p < 0.05.

The *Tet2*^−/−^ CHIP mouse model also recapitulated nonresolving AKI pathology. Twenty-eight days after ischemic injury, kidneys from *Tet2*^−/−^ mice maintained elevated mRNA and protein levels of kidney injury markers (Fig. 5b), increased mRNA for proinflammatory cytokines (Fig. 5c) and ongoing infiltration of leukocytes, as demonstrated by increased F4/80 mRNA and immunostaining for macrophages (Fig. 5d), increased mRNA for *Ly6g* (neutrophils), and *cd4* and *cd8* T cells (Extended Data Fig. 7). Isolated kidney macrophages expressed increased mRNA for proinflammatory genes and decreased mRNA of pro-recovery (‘M2’) genes (Fig. 5e). Kidneys from *Tet2*^−/−^ mice also maintained elevated mRNA expression of extracellular matrix genes (Fig. 5f) and developed significantly increased interstitial fibrosis, determined using Picrosirius red and Masson trichrome blue staining (Fig. 5g).

**Extended Data Fig. 8:**
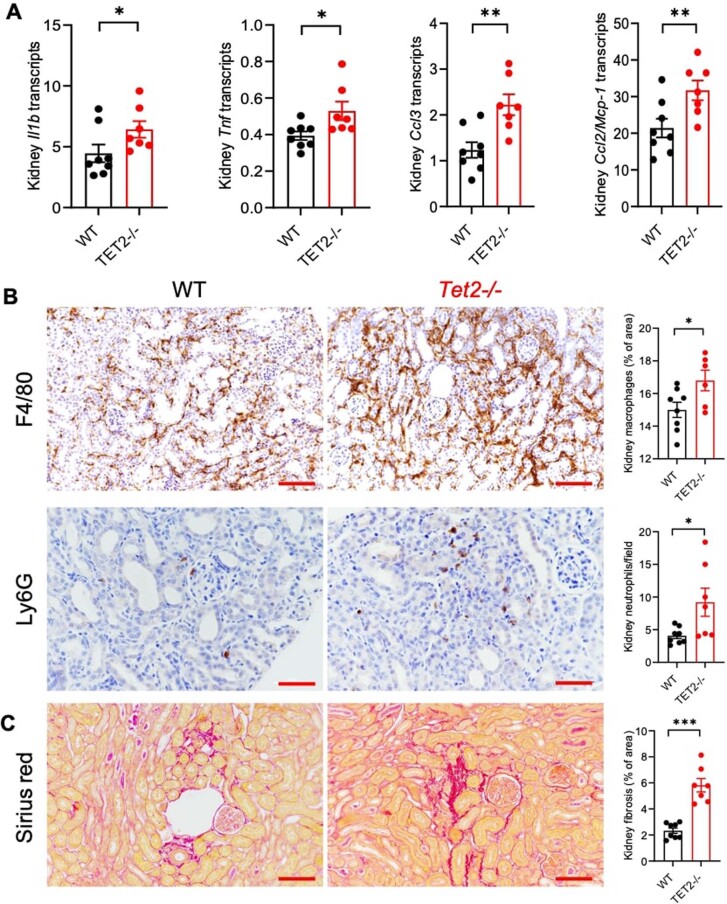
Increased inflammation, immune cell infiltration, and fibrosis is observed in *Tet2*^*-/*-^ mice after unilateral ureteral obstruction compared to WT control mice. **A)** mRNA of proinflammatory cytokines *Il1b, Tnf, Ccl3* and *Ccl2* in kidneys of WT and *Tet2*^*-/-*^ mice 7 days after UUO (n = 8 for WT and n = 7 for *Tet2*^*-/-*^) **B)** Representative figures and quantification of macrophage and neutrophil infiltration7 days after UUO (n = 8 for WT and n = 7 for *Tet2*^*-/-*^). **C)** Representative figure and quantification of interstitial fibrosis 7 days after UUO. (n = 8 for WT and n = 7 for *Tet2*^*-/-*^). Data were analyzed using 2-tailed Student’s t-tests and presented as mean ± SEM. *p < 0.05; **p < 0.01; scale bar = 50 µm.

We sought to recapitulate the kidney injury phenotype in an independent kidney injury model, UUO. Comparing mice that received a bone marrow transplant from WT mice to mice that received 20% *Tet2*^−/−^ bone marrow, the obstructed kidney of *Tet2*^−/−^ CHIP mice had increased mRNA expression of proinflammatory cytokines (*Il1b*, *Tnf*, *ccl3*, *ccl2*) at 7 days (Extended Data Fig. 8a), increased kidney macrophage (F4/80) and neutrophil (Ly6G) invasion (Extended Data Fig. 8b) and significantly increased interstitial fibrosis (Extended Data Fig. 8c).

**Fig. 6:**
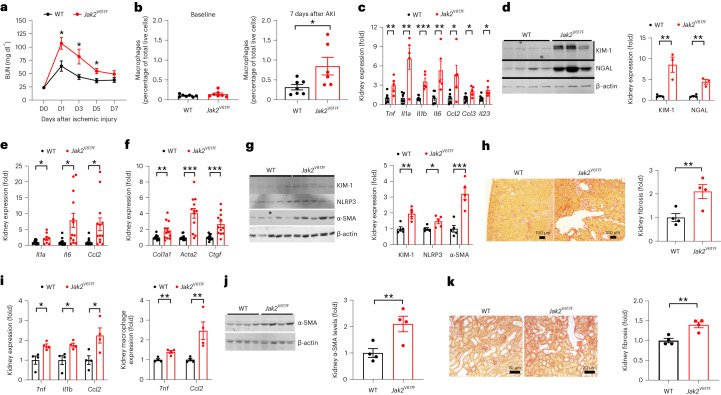
The response of hematopoietic-specific *Jak2*^V617F^ mice to acute and chronic kidney injury. **a**, Time course of BUN in response to ischemic kidney injury in WT and *Jak2*^V617F^ mice (*n* = 7 mice each). **b**, Quantification of kidney macrophages using flow cytometry in WT and *Jak2*^V617F^ mouse kidneys at baseline (*n* = 7 mice each) and 7 days after ischemic kidney injury (*n* = 7 in WT and 6 in *Jak2*^V617F^ mice). **c**, Kidney mRNA expression of the proinflammatory cytokines *Tnf*, *Il1a*, *Il1b*, *Il6*, *Ccl2*, *Ccl3* and *Il23* 7 days after ischemic injury in WT and *Jak2*^V617F^ mice (*n* = 5 mice each). **d**, KIM-1 and NGAL protein level 7 days after ischemic kidney injury in WT and *Jak2*^V617F^ mice (*n* = 4 mice each). **e**, Kidney mRNA of the proinflammatory cytokines *Il1a*, *Il6* and *Ccl2* 28 days after injury in WT and *Jak2*^V617F^ mice (*n* = 6 mice each). **f**, Kidney mRNA of the profibrotic factors *Col1a1*, *Acta2* and *Ctgf* 28 days after injury in WT and *Jak2*^V617F^ mice (*n* = 6 mice each). **g**, Quantification of kidney immunoreactive protein expression of KIM-1, NLRP3 and α-SMA 28 days after injury in WT and *Jak2*^V617F^ mice (*n* = 5 mice each). **h**, Representative images of kidney histology stained with Picrosirius red and quantification of kidney interstitial collagen expression 28 days after injury in WT and *Jak2*^V617F^ mice (*n* = 4 mice each). **i**, Macrophage mRNA of the proinflammatory cytokines *Tnf*, *I11b* and *Ccl2* in WT and *Jak2*^V617F^ mice 7 days after UUO (*n* = 4 mice each). **j**, Quantification of kidney α-SMA protein expression in the kidneys of WT and *Jak2*^V617F^ mice 7 days after UUO (*n* = 4 mice each). **k**, Representative images of kidney histology stained with Picrosirius red and quantification of kidney interstitial fibrosis in WT and *Jak2*^V617F^ mice 7 days after UUO (*n* = 4 mice each). Data were analyzed using a two-tailed Student’s *t*-test or two-way ANOVA followed by Tukey’s or Bonferroni’s post hoc tests and are presented as the mean ± s.e.m. *P < 0.05, **P < 0.01, ***P < 0.001. Source data

Finally, we performed kidney injury experiments in mice with a heterozygous gain of function mutation in *Jak2* that emulates the human CHIP hotspot mutation *JAK2* p.V617F (which we refer to as *Jak2*^V617F^ mice)^34,35^. Like the *Tet2*^*−/−*^ CHIP model, *Jak2*^V617F^ mice had higher BUN levels, greater macrophage infiltration, greater inflammatory gene expression and greater increases in the tubular injury markers KIM-1 and NGAL compared to WT mice 7 days after acute ischemic injury (Fig. 6a–d). Twenty-eight days after ischemic injury, the kidneys of *Jak2*^V617F^ mice showed sustained inflammatory gene expression, as demonstrated by higher mRNA levels of *Il1a*, *Il6* and *Ccl2* and higher protein levels of NLRP3 (Fig. 6e,g), as well as ongoing kidney injury (higher KIM-1 levels) compared to WT mice. At this time point, *Jak2*^V617F^ mouse kidneys also exhibited greater expression of fibrotic genes (*Col1a1*, *Acta2*, *Ctgf*; Fig. 6f), higher alpha smooth muscle actin (α-SMA) (Fig. 6g) and more severe fibrosis on histological examination (Fig. 6h) compared to WT mice. We also evaluated the UUO injury model in *Jak2*^V617F^ mice and observed increased mRNA of proinflammatory cytokines in the whole-kidney specimen and in isolated renal macrophages, increased α-SMA protein expression and more interstitial fibrosis 7 days after injury compared to WT mice (Fig. 6i–k).

## Discussion

By integrating large-scale human genetic data from five human epidemiological studies, four distinct in vivo models, as well as MR and single-cell studies, we identified that CHIP is associated with AKI and poor kidney outcomes after kidney injury via an aberrant renal macrophage inflammatory response. The roughly twofold increased risk of AKI progressing to dialysis and nonresolving AKI we observed in non-*DNMT3A*-CHIP clones was similar in magnitude to that observed for CHIP and cardiovascular disease. This association was independent of age, sex, smoking status, baseline eGFR and presence of diabetes and hypertension, with effect estimates remarkably consistent across cohorts despite notable differences in baseline characteristics and AKI incidence. We observed consistent results in a MR analysis, suggesting a causal role for CHIP in AKI. The association we observed between CHIP and AKI was in marked contrast with all previous efforts to identify genetic factors associated with AKI, which have focused on common, inherited genetic variants.

The association between CHIP and incident AKI events may reflect increased predisposition to AKI, greater severity of AKI or impaired recovery from AKI. To distinguish these mechanisms, we assessed short-term and long-term outcomes after AKI according to CHIP status in participants enrolled in the ASSESS-AKI study. Although limited by the small sample size, we showed that non-*DNMT3A*-CHIP was associated with a nonresolving AKI pattern, findings that were replicated in an independent cohort consisting of participants in the BioVU repository. Additionally, as ASSESS-AKI participant CHIP status was ascertained using a targeted sequencing method that is highly sensitive to small CHIP clones, we conducted a sensitivity analysis examining only large clones with a VAF ≥ 10%. We identified that large CHIP clones were associated both with a nonresolving AKI pattern and with higher rates of kidney failure over 5 years of study follow-up. These data suggest that non-*DNMT3A*-CHIP is associated with impaired recovery from AKI in humans.

While CHIP as a heterogenous entity is associated with several age-related diseases, several studies reported positive findings for non-*DNMT3A*-CHIP and smaller or null effect sizes for *DNMT3A*-CHIP^9,17,36,37^. In line with this, the newly developed clonal hematopoiesis risk score, which stratifies the risk of CHIP progression to myeloid cancer and of developing CHIP-associated comorbidities including cardiovascular and kidney disease, accords more points for non-*DNMT3A*-CHIP compared to *DNMT3A-*CHIP^38^.

To further demonstrate that non-*DNMT3A*-CHIP is causally associated with AKI and delineate disease mechanisms, we focused on CHIP caused by inactivating mutations in *TET2* and *JAK2* p.V617F, which made up more than 35% of non-*DNMT3A*-CHIP cases in our study and are each independently associated with AKI risk. After experimental ischemic or obstructive kidney injury, *Tet2-*CHIP and *Jak2*^V617F^-CHIP mice displayed more severe kidney injury and impaired recovery after kidney injury when compared to mice with intact *Tet2* and *Jak2*, respectively, as evidenced by higher serum levels of markers of impaired kidney function and injury (creatinine and BUN; KIM-1 and NGAL), as well as structural kidney damage, including higher and more pronounced initial tubular injury and kidney interstitial fibrosis 28 days after kidney injury.

Greater local inflammation by infiltrating proinflammatory macrophages appeared to underlie the worse outcomes after kidney injury in *Tet2*-CHIP and *Jak2*^V617F^-CHIP mice. We observed elevated levels of several inflammatory cytokines and chemokines in the kidneys of *Tet2*-CHIP and *Jak2*^V617F^-CHIP mice, primarily derived from kidney-infiltrating macrophages. Additionally, we showed that infiltrating *Tet2*^−/−^ renal macrophages but not *Tet2*^+/+^ renal macrophages had heightened NLRP3 inflammasome activation and IL-1β production, as well as global inflammatory and profibrotic gene upregulation in the scRNA-seq analyses. We and others previously observed that *Tet2*^−/−^-derived macrophages are similarly hyperactivated in other tissue contexts^9,10,15,17,18,39^. Overall, our study suggests that CHIP has an inhibitory role in recovery after AKI because of increased proinflammatory signaling by mutated infiltrating macrophages.

Limitations of our study include an inability to examine the relevance of CHIP in all subtypes of AKI in the prospective cohort analyses. While *Tet2*-CHIP and *Jak2*^V617F^-CHIP conferred a similar hyperinflammatory phenotype in both our ischemic and obstructive mouse models, we did not study any intrarenal kidney injury models. The impact of CHIP in glomerulonephritis and drug-induced AKI, for example, requires a separate study. Additionally, CHIP and AKI are both associated with age; although we adjusted for age and age^2^ in our prospective cohort analyses to model the shape of these associations (as shown in Extended Data Fig. 1a,c), it is possible that collinearity of these variables may have resulted in residual confounding of our risk estimates. However, such collinearity is equally present in *DNMT3A* and non-*DNMT3A-*CHIP genes, so the fact that these CHIP subsets have divergent clinical consequences suggests that residual confounding is not driving the association.

In conclusion, CHIP is associated with an elevated risk of AKI specifically through the promotion of renal macrophage inflammation. CHIP affects 10–20% of individuals aged 65 and over^29,40^, an age group that is especially vulnerable to AKI. Through targeting of the NLRP3 inflammasome or downstream mediators, CHIP may be a modifiable risk factor for AKI and progression to end-stage kidney disease (ESKD).

## Methods

### CHIP ascertainment

Acquired DNA mutations meeting the established criteria for CHIP were identified as described previously in the UKB whole-exome sequencing dataset^40^ (UKB, *n* = 428,793) and in whole-genome sequencing data from the TOPMed sequencing initiatives for the ARIC (*n* = 10,570) and CHS (*n* = 2,790) cohorts^29^. For the ASSESS-AKI and BioVU cohorts, CHIP was assessed using a targeted sequencing panel (Twist Bioscience). Putative somatic variants meeting previously defined criteria for CHIP^9^ were identified using the somatic variant caller Mutect2 and filtered using established filtering criteria^40^. Subgroups of *DNMT3A*-CHIP and non-*DNMT3A*-CHIP were defined based on the identity of the mutated gene with the largest VAF per person. Additionally because CHIP status in ASSESS-AKI and BioVU was ascertained using targeted sequencing, a method that that is highly sensitive to small clones compared to whole-exome and whole-genome-based detection^40^, a ‘large CHIP’ subgroup was defined as CHIP variants with a VAF ≥ 10%.

### Outcome ascertainment

For the analyses in the UKB, ARIC and CHS, incident AKI was ascertained as hospitalizations or deaths with an International Statistical Classification of Diseases and Related Health Problems (ICD) code for AKI in any position (ICD-9-CM code 584.x or ICD-10-CM code N17.x)^27,28^. In CHS, the following additional ICD-9 codes were included in the AKI definition: 788.9 (uremia); 586 (renal failure, not otherwise specified); 39.95 (hemodialysis); 54.98 (peritoneal dialysis); and V56.8 (peritoneal); all incident AKI events underwent manual chart review to ensure they met the definition for AKI^27^. An additional phenotype of severe AKI requiring dialysis (AKI-D) was defined in the UKB as any AKI event with a procedure code for dialysis (OPCS4 X40.x) within 30 days of AKI. Individuals with a baseline eGFR lower than 15 ml per min per 1.73 m^2^ (baseline creatinine greater than 3.0 mg dl^−1^ in CHS) or documented ESKD, as well as individuals without baseline kidney function measurement, were excluded from all analyses. In all three cohorts, the first AKI event was considered as the incident event and no subsequent events were evaluated. Individuals with known AKI before enrollment were excluded from the UKB analysis because events are noted only once per person (a limitation of UKB data), whereas previous AKI was not an exclusion criterion in the TOPMed cohorts.

For the analyses in the ASSESS-AKI cohort, we used the primary study authors’ definitions when examining the following phenotypes and outcomes: AKI cases and non-AKI controls^30^; nonresolving AKI and resolving AKI cases (resolving AKI defined as a decrease in serum creatinine concentration of 26.5 mmol l^−1^ or 25% or more from maximum in the first 72 h after AKI diagnosis); nonresolving AKI defined as any AKI not meeting the definition for resolving AKI^5^; and primary ASSESS-AKI study outcome (that is, halving of the estimated eGFR or ESKD)^5^. For the replication analyses in the BioVU cohort, we designed a case-control study to investigate the association between CHIP and recovery after AKI. AKI cases were defined using modified Kidney Disease: Improving Global Outcomes criteria based on an increase of 0.3 mg dl^−1^ or more in serum creatinine or an increase of 50% or more in serum creatinine above a baseline value. The baseline serum creatinine value was an outpatient, nonemergency department measurement within 7–365 days before the index admission. For each patient, only AKI cases occurring after the date of CHIP ascertainment were retained in the analytical sample. As in ASSESS-AKI, resolving AKI was defined as a decrease in serum creatinine levels of 0.3 mg dl^−1^ or more or 25% or more from the maximum in the first 72 h after AKI diagnosis. Nonresolving AKI was defined as all AKI cases not meeting the definition of resolving AKI. If participants were discharged from hospital before 72 h after AKI diagnosis, then the last serum creatinine measurement before hospital discharge was used to determine the criteria for resolving or nonresolving AKI. All participants with resolving AKI had to have a sustained decrease in serum creatinine concentration during the 72-h time window. Finally, patients with AKI requiring dialysis during admission were classified as nonresolving AKI cases regardless of their inpatient creatinine trajectory.

### Statistical analysis of prospective cohort studies

For the prospective cohort studies, statistical analyses were performed using R v.4.2.1. Associations of CHIP with incident AKI were assessed using Cox proportional hazards regression while adjusting for age, age^2^, sex, baseline eGFR, baseline smoking status, diabetes and hypertension, and either ten principal components of ancestry (UKB) or self-reported ethnicity (TOPMed cohorts). In the subgroup analyses in the UKB, baseline CKD was defined as a baseline eGFR less than 60 ml per min per 1.73 m^2^ or an ICD code for CKD stages 3–5 dated before enrollment. Individuals were censored at death, ESKD or end of study follow-up in the incident AKI analyses; results were meta-analyzed using a fixed-effect model. For ASSESS-AKI, cross-sectional outcomes were analyzed using chi-squared tests and logistic regression analyses adjusted for age, sex, baseline creatinine, AKI stage, smoking status and ethnicity, and history of diabetes, hypertension and cardiovascular disease; the incident primary outcome was assessed using Cox proportional hazards regression adjusting for the same set of covariates. For the BioVU analyses, covariates included age, sex, ethnicity, baseline creatinine, history of diabetes and history of hypertension.

#### MR

We performed two-sample MR analyses to investigate the effect of genetically predicted CHIP risk on AKI risk using summary data from a large discovery genome-wide association study (GWAS) of CHIP in 454,803 individuals in the UKB and 173,585 participants in the Geisinger MyCode Community Health Initiative^41^. CHIP risk genetic instruments (*P* < 5 × 10^−8^) were independent (clumped at an *r*^2^ of less than 0.1). Estimates of the single-nucleotide polymorphism (SNP) outcome association between each CHIP-associated variant and AKI were obtained using data from ASSESS-AKI^42^ and BioVU, which is linked to de-identified electronic health records. We excluded variants in the telomerase reverse transcriptase (*TERT*) gene because these are associated with several comorbidities and are likely pleiotropic^43^. The primary analysis was conducted using the conventional multiplicative random effects IVW estimator. Outliers were investigated using standard approaches including Cook’s distance, studentized residuals and residual versus leverage plots. Sensitivity analyses were conducted using outlier-robust approaches including weighted median, weighted mode and MR-Egger methods. MR-Egger was particularly important given a priori concerns of pleiotropy because it allows for simultaneous estimation of pleiotropic and causal effects. The SNP heritability for overall CHIP in the discovery GWAS was approximately 4% and the independent SNPs used as genetic proxies for CHIP risk explained 0.6% of the variance for CHIP after excluding the *TERT* SNPs.

### Animals

To determine the effect of hematopoietic *Tet2* deficiency in murine models of AKI, bone marrow transplants were performed as reported previously^44^. Briefly, recipient mice were lethally irradiated with 9 Gy using a cesium γ source. Bone marrow cells were collected from syngeneic donor femurs and tibias. Recipient mice received a total of 5 × 10^6^ bone marrow cells in 0.2 ml medium through retro-orbital injection. Mice received either 100% WT bone marrow or 80% WT and 20% bone marrow from either mice with hematopoietic stem cell-specific *Tet2* deletion (*Vav1-iCre;Tet2*^−/−^). All donor mice had the CD45.2 isotype and recipient mice had the CD45.1 isotype (Extended Data Fig. 4a). C57BL/6 WT mice, C57B6/J *Tet2*^*loxP*/*loxP*^ mice with *loxP* sites flanking *Tet2* exon 3 (strain no. 017573, The Jackson Laboratory) and C57B6/J Vav1-iCre mice that enables conditional gene knockout in hematopoietic stem cells (strain no. 008610) were bred at Queen’s University (approved University Animal Care Committee protocol 2021–2128) and provided under material transfer agreement to R. Harris (Vanderbilt University). C57BL/6 Cd45.1Pep Boy mice were obtained from The Jackson Laboratory (strain no. 002014). Flow cytometry was used to determine the effectiveness of engraftment in the chimeras and assessment of clonal expansion of the hematopoietic cells carrying the *Tet2* deletion. Injury studies were performed 6–8 weeks later, when mutant hematopoietic cells were expanded to approximately 60% of the total (Extended Data Fig. 4b)^9,10,34^. Male mice were used for all studies involving *Tet2*^−/−^ mice.

For the *Jak2* mouse model studies, C57BL/6 mice with an inducible mutation in *Jak2*^V617F^-MX1-Cre^+^ were obtained from The Jackson Laboratory (strain no. 037558). At 8–9 weeks of age, heterozygous *Jak2*^V617F^ mice were injected with 20 µg g^−1^ body weight of polyinosinic:polycytidylic acid (poly I:C) 3 days a week for 2 weeks before the injury studies. Male and female mice were used for studies involving *Jak2*^V617F^ mice.

#### Injury models

For the acute ischemia–reperfusion studies, mice were anesthetized and subjected to unilateral kidney vascular clamping for 32 min with simultaneous contralateral nephrectomy. The vascular clamp was then removed and mice were monitored until they recovered from the anesthesia^45^. For chronic ischemia–reperfusion studies, the left kidney was subjected to 32 min of ischemia and the right kidney was not removed until after operative day 8. For UUO, mice were anesthesized and the left ureter was ligated. Mice were then monitored until they recovered from the anesthesia^46^.

### Quantitative immunofluorescence and IHC staining

Kidney tissue was immersed in fixative containing 3.7% formaldehyde, 10 mM sodium metaperiodate, 40 mM phosphate buffer and 1% acetic acid. The tissue was dehydrated through a graded ethanol series, embedded in paraffin, sectioned (4 μm) and mounted on glass slides. For immunofluorescence (IF) staining, sections were incubated for two rounds of staining overnight at 4 °C. Anti-rabbit or anti-mouse IgG-horseradish peroxidase was used as a secondary antibody. Each round was followed by tyramide signal amplification with the appropriate fluorophore (Alexa Fluor 488 tyramide, Alexa Flour 647 tyramide or Alexa Fluor 555 tyramide, Tyramide SuperBoost Kit with Alexa Fluor Tyramides, Invitrogen) according to the manufacturer’s protocols. 4′,6-Diamidino-2-phenylindole was used as a nuclear stain. Sections were viewed and imaged with a Nikon TE300 fluorescence microscope and spot-cam digital camera (Diagnostic Instruments), followed by quantification using Image J (NIH).

### Immunoblotting analysis

Whole-kidney tissue was homogenized with lysis buffer containing 10 mmol l^−1^ Tris-HCl (pH 7.4), 50 mmol l^−1^ NaCl, 2 mmol l^−1^ EGTA, 2 mmol l^−1^ EDTA, 0.5% Nonidet P-40, 0.1% SDS, 100 μmol l^−1^ Na_3_VO_4_, 100 mmol l^−1^ NaF, 0.5% sodium deoxycholate, 10 mmol l^−1^ sodium pyrophosphate, 1 mmol l^−1^ phenylmethylsulfonyl fluoride, 10 μg ml^−1^ aprotinin and 10 μg ml^−1^ leupeptin and centrifuged at 15,000*g* for 20 min at 4 °C. The BCA Protein Assay Kit (Thermo Fisher Scientific) was used to measure protein concentration. Immunoblotting (IB) was quantitated with Image J. Raw images are presented in the Source data files.

### Antibodies

Antibodies used for IB, IF, IHC and flow cytometry were: NLRP3 (1:500 dilution for IB and 1:100 for IF, cat. no. PAS079740, Thermo Fisher Scientific); IL-1β (1:300 dilution for IB and 1:50 for IF, cat. no. P420B, Thermo Fisher Scientific); NGAL (1:500 dilution for IB, cat. no. AF1857, R&D Systems); KIM-1 (1:500 dilution for IB, cat. no. AF1817, R&D Systems); β-actin (1:1,000 dilution for IB, cat. no. 4967, Cell Signaling Technology), α-SMA (1:1,000 dilution for IB, cat. no. ab21027, Abcam); CD68 (1:100 dilution for IF, cat. no. 125212, Abcam); CD45 (0.1 mg ml^−1^ for FC, cat. no. 103149, BioLegend); CD45.1 (0.2 mg ml^−1^ for FC and 1:50 dilution for IF, cat. no. 17-0453-82, Thermo Fisher Scientific); CD45.2 (0.2 mg ml^−1^ for FC and 1:50 dilution for IF, cat. no. 14-0454-82, Thermo Fisher Scientific); F4/80 (1:100 dilution for IHC, cat. no. MCA497, Bio-Rad Laboratories); and Ly6G (1:2,000 dilution for IHC, cat. no. ab238132, Abcam).

### qPCR

Total RNAs from kidneys or cells were isolated using TRIzol reagent (Invitrogen). The SuperScript IV First-Strand Synthesis System kit (Invitrogen) was used to synthesize complementary DNA from equal amounts of total RNA from each sample. qPCR with reverse transcription was performed using TaqMan real-time PCR (cat. no. 7900HT, Applied Biosystems). The Master Mix and all gene probes were also sourced from Applied Biosystems. The probes used in the experiments included mouse *Tnf* (Mm99999068), *Il1b* (Mm00434228), *Il6* (Mm00446190), *Ccl3* (Mm00441258), *Ccl2* (Mm00441242), *Il23a* (Mm00518984), *Col1a1* (Mm00801666), *Col3a1* (Mm01254476), *Col4a1* (Mm01210125), *Acta2* (Mm01546133), *Fn* (Mm01256744), *Tgfb1* (Mm00441726), *Ccn2* (*Ctgf*) (Mm01192932), *Havcr1* (KIM-1 Mm00506686)*, Lcn2* (*Ngal*) (Mm01324470)*, Vim* (Mm01333430)*, cd68* (Mm03047343), *il10* (Mm004391)*, CD209a* (Mm00460067)*, cd163* (Mm00474091)*, Mrc1* (*CD206*) (Mm01329362); *Gapdh* (Mm99999915) was used as a normalizer. Real-time PCR data were analyzed using the 2-ΔΔ^Ct^ method to determine the fold difference in expression.

Picrosirius red stain was performed according to the protocol provided by the manufacturer (Sigma-Aldrich).

### Kidney tubular injury score

Periodic acid–Schiff-stained slides were used to evaluate the tubular injury score. Sections were assessed by counting the percentage of tubules that displayed cell necrosis, loss of brush border, cast formation and tubule dilation as follows: 0, normal; 1, less than 10%; 2, 10–25%; 3, 26–50%; 4, 51–75%; 5, more than 75%. Five fields from each outer medulla were evaluated and scored in a blinded manner by two observers and the results were averaged.

### Statistical analyses for the animal experiments

For the animal experiments, statistical analyses were performed with Prism 9 (GraphPad Software). Data are presented as the mean ± s.e.m. Data were analyzed using a two-tailed Student’s *t*-test or two-way ANOVA followed by Tukey’s or Bonferroni’s post hoc tests. *P* < 0.05 was considered significant. For each set of data, at least six different animals were examined for each condition. Collection, analysis and interpretation of the data were conducted by at least two independent investigators.

### scRNA-seq analyses

#### Sample preparation

WT and *Tet2*^*−/−*^ mouse kidneys were removed 3 days after ischemic injury. The sample was enriched for CD45^+^ (hematopoietic) cells using anti-mouse CD45 MicroBeads and MACS columns (cat. no. 130-110-443, Miltenyi Biotec) according to the manufacturer’s protocol. Samples were then submitted for processing using the 10X Genomics platform (more than 20,000 cells per sample).

#### Preprocessing

A standard Cell Ranger workflow was applied to demultiplex raw base call files and align sequencing reads (10X Genomics Cell Ranger v.1.1.0). The Cell Ranger outputs were converted to Seurat format for further processing (Seurat v.4.9.9.9060). Ambient RNA was removed using SoupX v.1.6.2 (https://github.com/constantAmateur/SoupX)^47^. Cells of poor sequencing quality (fewer than 200 genes, fewer than 800 transcripts and more than 15% mitochondrial reads) were removed from the dataset. The dataset was then normalized and scaled using the sctransform function in Seurat; doublets were removed using the DoubletFinder R package v.2.0.3 (https://github.com/chris-mcginnis-ucsf/DoubletFinder)^48^. Transcripts for mitochondrial, ribosomal, sex-specific and red blood cell genes were removed. Finally datasets were integrated using the Harmony R package v.0.1.1 (https://github.com/immunogenomics/harmony) to minimize batch effects^49^.

#### Cell type annotation

Cell types were assigned by transferring anchors from an existing mouse kidney atlas^50^. The following cell types were identified: macrophage; neutrophil; dendritic cell; ‘native cell’ (tissue-resident macrophage); T cell; endothelial cell (including glomerular endothelial cell); podocyte; pericyte; mesangial cell; proximal tubule epithelial cell; loop of Henle epithelial cell; distal convoluted tubule epithelial cell; collecting duct or connecting tubule epithelial cell; collecting duct principal cell; renal alpha-intercalated cell; and renal beta-intercalated cell. (The counts for each cell type listed in Supplementary Table 3.) Cell annotations were further refined based on clustering in UMAP projections: cells annotated as ‘macrophage’ but that fell within a cluster where another cell type predominated were removed from the dataset. Finally, neighboring clusters of macrophages and ‘native cells’ (tissue-resident macrophages) were collapsed into a single ‘macrophage’ group for subsequent analyses.

### Differential gene expression analyses

To prepare the data for differential gene expression (DGE) analysis with DESeq2, files were first converted from Seurat format to a Python-compatible 10X format, which enabled grouping of cells in the dataset into meta-cells using Metacell-2 in Python (v.0.8.0)^51^. The reads within each meta-cell were aggregated by summing transcripts with SingleCellExperiment v.1.22.0 in Bioconductor^52^. DGE was calculated for each gene using negative binomial generalized linear model fitting and a Wald test using DESeq2 v.1.40.2. Only genes with three or more transcripts were evaluated.

### Pathway analyses

GSEA was performed using clusterProfiler (v.4.8.1)^53,54^, with ontology limited to ‘Biological Pathways’.

### Cell–cell communication analyses

Signaling between cell types in the data was characterized using the CellChat R package v.1.6.1 (https://github.com/sqjin/CellChat)^55^.

### Study approval

Access to the UKB dataset was provided under application no. 43397. Local approval for secondary analyses of the data was obtained from the Vanderbilt University Medical Center Institutional Review Board (nos. 210728, 210270, 220035). Written informed consent was obtained from all participants for the purposes of this study. The Vanderbilt University Medical Centre animal care committee approved all animal study procedures.

### Reporting summary

Further information on research design is available in the Nature Portfolio Reporting Summary linked to this article.

## Online content

Any methods, additional references, Nature Portfolio reporting summaries, source data, extended data, supplementary information, acknowledgements, peer review information; details of author contributions and competing interests; and statements of data and code availability are available at 10.1038/s41591-024-02854-6.

### Supplementary information

Supplementary Information Supplementary Tables 1–3.

Reporting Summary

### Source data

Source Data Fig. 3 Unprocessed immunoblots.

Source Data Fig. 5 Unprocessed immunoblots.

Source Data Fig. 6 Unprocessed immunoblots.

## Data Availability

CHIP calls for the UKB participants have been returned to the UKB Access Management System (AMS) and will be available to all registered researchers once processed by the UKB AMS team^40^. CHIP calls and phenotypes for the TOPMed cohorts used in this analysis are available through restricted access via the database of Genotypes and Phenotypes (accession nos. phs001211 for ARIC and phs001368 for CHS). Data from the Assessment, Serial Evaluation, and Subsequent Sequelae in Acute Kidney Injury (V2; 10.58020/js2k-9d54) reported here are available for request at the NIDDK Central Repository website, Resources for Research (https://repository.niddk.nih.gov/). Data for the BioVU cohort are available at 10.5281/zenodo.8377764. Source data are provided with this paper.
