# Supplementary material for: Clonal hematopoiesis of indeterminate potential is associated with acute kidney injury
Source: Nat Med. 2024 Mar 7;30(3):810–7. doi: 10.1038/s41591-024-02854-6 (PMC10957477; doi:10.1038/s41591-024-02854-6)
Supplement: Supplementary file 1 — Supplementary Tables 1–3. [file 41591_2024_2854_MOESM1_ESM.pdf]

nature medicine

Article https://doi.org/10.1038/s41591-024-02854-6

Clonal hematopoiesis of indeterminate potential is associated with acute kidney injury

In the format provided by the authors and unedited

Supplementary Table 1. Baseline characteristics for prospective cohort studies

	UK Biobank (N = 428,793)		ARIC (N = 10,570)		CHS (N = 2,790)	
	CHIP	*No CHIP*	*CHIP*	*No CHIP*	*CHIP*	*No CHIP*
Participants (N)	14,552	414,241	743	9,827	404	2,386
Age (years; mean ± SD)	61 ± 7	56 ± 8	60 ± 6	58 ± 6	75 ± 6	74 ± 6
Male sex (%)	47	46	48	45	47	43
eGFR (ml/min/1.73m^2; mean ± SD)	91 ± 15	95 ± 14	93 ± 16	96 ± 15	66 ± 16	69 ± 16
Diabetes (%)	2	1	10	10	19	16
Hypertension (%)	8	6	4	4	47	46
Smoking (%)	54	41	62	57	54	55

Supplemental Table 2. Mendelian randomization estimates for the effect of genetically-predicted CHIP risk on AKI risk

CHIP → AKI	*Inverse Variance Weighted*		*MR-Egger*	
	Beta (SE)	OR (95%CI)	Beta (SE)	OR (95%CI)
ASSESS-AKI	0.333 (0.200)	1.40 (0.94-2.06)	0.295 (0.673)	1.34 (0.36-5.03)
BioVU	0.178 (0.114)	1.20 (0.96-1.49)	0.670 (0.332)	1.95 (1.01-3.75)
**Meta-analysis**	**0.216 (0.099)**	**1.24 (1.02-1.51)**	**0.597 (0.298)**	**1.82 (1.01-3.26)**

Supplementary Table 3. Cell counts for single-cell analyses

Cell type	Count *Tet2*$^{-/-}$	% *Tet2*$^{-/-}$	Count WT	% WT
macrophage	18220	76.2	17165	75.5
neutrophil	2221	9.3	41	0.2
"native cell" (tissue-resident macrophage)	1145	4.8	1072	4.7
T-cell	766	3.2	1569	6.9
dendritic cell	379	1.6	617	2.7
proximal tubule epithelial cell	336	1.4	1330	5.9
endothelial cell	254	1.1	192	0.8
collecting duct principal cell	191	0.8	154	0.7
distal convoluted tubule epithelial cell	111	0.5	240	1.1
loop of Henle epithelial cell	98	0.4	104	0.5
collecting duct / connecting tubule epithelial cell	79	0.3	80	0.4
renal beta-intercalated cell	54	0.2	69	0.3
podocyte	34	0.1	38	0.2
renal alpha-intercalated cell	22	0.1	29	0.1
pericyte	7	0.03	14	0.06
mesangial cell	4	0.02	12	0.05
Total	23921	100	22726	100
