# Supplementary material for: Clonal hematopoiesis of indeterminate potential is associated with acute kidney injury
Source: Nat Med. 2024 Mar 7;30(3):810–7. doi: 10.1038/s41591-024-02854-6 (PMC10957477; doi:10.1038/s41591-024-02854-6)
Supplement: Supplementary file 2 — Reporting Summary [file 41591_2024_2854_MOESM2_ESM.pdf]

nature portfolio

Corresponding author(s): Alexander Bick

Last updated by author(s): January 21, 2024

Reporting Summary

Nature Portfolio wishes to improve the reproducibility of the work that we publish. This form provides structure for consistency and transparency in reporting. For further information on Nature Portfolio policies, see our Editorial Policies and the Editorial Policy Checklist.

Statistics

For all statistical analyses, confirm that the following items are present in the figure legend, table legend, main text, or Methods section.

n/a	Confirmed	
☐	☒	The exact sample size (*n*) for each experimental group/condition, given as a discrete number and unit of measurement
☐	☒	A statement on whether measurements were taken from distinct samples or whether the same sample was measured repeatedly
☐	☒	The statistical test(s) used AND whether they are one- or two-sided<br>*Only common tests should be described solely by name; describe more complex techniques in the Methods section.*
☐	☒	A description of all covariates tested
☐	☒	A description of any assumptions or corrections, such as tests of normality and adjustment for multiple comparisons
☐	☒	A full description of the statistical parameters including central tendency (e.g. means) or other basic estimates (e.g. regression coefficient) AND variation (e.g. standard deviation) or associated estimates of uncertainty (e.g. confidence intervals)
☐	☒	For null hypothesis testing, the test statistic (e.g. *F*, *t*, *r*) with confidence intervals, effect sizes, degrees of freedom and *P* value noted<br>*Give P values as exact values whenever suitable.*
☒	☐	For Bayesian analysis, information on the choice of priors and Markov chain Monte Carlo settings
☒	☐	For hierarchical and complex designs, identification of the appropriate level for tests and full reporting of outcomes
☐	☒	Estimates of effect sizes (e.g. Cohen's *d*, Pearson's *r*), indicating how they were calculated

Our web collection on statistics for biologists contains articles on many of the points above.

Software and code

Policy information about availability of computer code

Data collection	No software was used to collect data.
Data analysis	For the prospective cohort studies, statistical analyses were performed using R version 4.2.1. For animal experiments, statistical analyses were performed with GraphPad Prism 9.

For manuscripts utilizing custom algorithms or software that are central to the research but not yet described in published literature, software must be made available to editors and reviewers. We strongly encourage code deposition in a community repository (e.g. GitHub). See the Nature Portfolio guidelines for submitting code & software for further information.

Data

Policy information about availability of data

All manuscripts must include a data availability statement. This statement should provide the following information, where applicable:

- Accession codes, unique identifiers, or web links for publicly available datasets
- A description of any restrictions on data availability
- For clinical datasets or third party data, please ensure that the statement adheres to our policy

CHIP calls for UKB participants have been returned to the UKB Access Management System (AMS) and will be available to all registered researchers once processed by the UKB AMS team. CHIP calls and phenotypes for TOPMed cohorts used in this analysis are available through restricted access via the dbGaP (accession numbers: phs001211.v4.p3 for ARIC and phs001368.v4.p2 for CHS). Data from the Assessment, Serial Evaluation, and Subsequent Sequelae in Acute Kidney Injury

[(V2)/https://doi.org/10.58020/js2k-9d54] reported here are available for request at the NIDDK Central Repository (NIDDK-CR) website, Resources for Research (R4R), https://repository.niddk.nih.gov/. Data for the BioVU cohort are available at doi: 10.5281/zenodo.8377764.

Research involving human participants, their data, or biological material

Policy information about studies with human participants or human data. See also policy information about sex, gender (identity/presentation), and sexual orientation and race, ethnicity and racism.

Reporting on sex and gender	The prospective analyses were adjusted for biological sex as a binary variable. The Tet2 mouse studies were conducted in male mice, while the Jak2 mouse studies were conducted in both male and female mice.
Reporting on race, ethnicity, or other socially relevant groupings	Prospective analyses in the UK Biobank were adjusted for 10 principal components of genetic ancestry. Prospective anaylses in the TOPMed and ASSESS-AKI cohorts were adjusted for self-reported race or ethnicity as a categorical variable.
Population characteristics	Population characteristics differ across cohorts studied in this manuscript and are described in Table 1 of the manuscript.
Recruitment	N/A – we did not recruit the patients
Ethics oversight	The UK Biobank study approval committee, the TOPMed Kidney Working Group, the ARIC and CHS manuscript proposal committees, the ASSESS-AKI NIDDK repository, and Vanderbilt University Medical Center Institutional Review Board (IRB# 210728, 210270, 220035). Informed consent was obtained from all participants for this work.

Note that full information on the approval of the study protocol must also be provided in the manuscript.

Field-specific reporting

Please select the one below that is the best fit for your research. If you are not sure, read the appropriate sections before making your selection.

☒ Life sciences ☐ Behavioural & social sciences ☐ Ecological, evolutionary & environmental sciences

For a reference copy of the document with all sections, see nature.com/documents/nr-reporting-summary-flat.pdf

Life sciences study design

All studies must disclose on these points even when the disclosure is negative.

Sample size	All samples with available DNA and/or CHIP calls were utilized in the epidemiologic analyses.
Data exclusions	No data were excluded.
Replication	For the epidemiologic studies, the findings were consistent across 3 cohorts studied. For the mouse studies, at least 3 biologic replicates were used for each finding (specific number indicated in the Figure legends), and all attempts at replication were successful.
Randomization	Allocation of mice to receive CHIP-deficient or wild-type bone marrow was random.
Blinding	Interpretation of subjective metrics (e.g., histologic findings) was conducted in a blinded fashion.

Reporting for specific materials, systems and methods

We require information from authors about some types of materials, experimental systems and methods used in many studies. Here, indicate whether each material, system or method listed is relevant to your study. If you are not sure if a list item applies to your research, read the appropriate section before selecting a response.

Materials & experimental systems

n/a	Involved in the study	
☐	☒	Antibodies
☒	☐	Eukaryotic cell lines
☒	☐	Palaeontology and archaeology
☐	☒	Animals and other organisms
☒	☐	Clinical data
☒	☐	Dual use research of concern
☒	☐	Plants

Methods

n/a	Involved in the study	
☒	☐	ChIP-seq
☒	☐	Flow cytometry
☒	☐	MRI-based neuroimaging

Antibodies

Antibodies used	Antibodies used for immunoblotting (IB), immunofluorescence (IF), immunohistochemistry (IHC), and flow cytometry (FC) were: NLRP3 (ThermoFisher Cat #PAS079740, 1:500 dilution for IB and 1:100 for IF), IL-1β (ThermoFisher Cat #P420B, 1:300 dilution for IB and 1:50 for IF), NGAL (R&D Systems Cat #AF1857, 1:500 dilution for IB), KIM-1 (R&D Systems Cat #AF1817, 1:500 dilution for IB), β-actin (Cell Signaling Technology Cat #4967, 1:1000 dilution for IB), α-SMA (Abcam Cat #ab21027, 1:1000 dilution for IB), CD68 (Abcam Cat #ab125212, 1:100 dilution for IF), CD45 (Biolegend Cat # 103149, 0.1 mg/ml for FC), CD45.1 (ThermoFisher Cat #17-0453-82, 0.2 mg/ml for FC and 1:50 dilution for IF), CD45.2 (ThermoFisher Cat #14-0454-82, 0.2 mg/ml for FC and 1:50 dilution for IF), F4/80 (BioRad Cat #MCA497, 1:100 dilution for IHC), and Ly6G (Abcam Cat #ab238132, 1:2000 dilution for IHC).
Validation	Validation of antibody specificity and relevant citations for all of these antibodies is presented in the manufacturers' information on their websites, which are easily searchable.

Animals and other research organisms

Policy information about studies involving animals; ARRIVE guidelines recommended for reporting animal research, and Sex and Gender in Research

Laboratory animals	C57BL/6 wild-type mice, C57B6/J Tet2f/f mice with loxP sites flanking Tet2 exon 3 (Jackson Laboratories strain number: 017573), and C57B6/J Vav1-iCre mice that enables conditional gene knockout in hematopoietic stem cells (strain number: 008610) were bred at Queen's University and provided under material transfer agreement to Dr. Raymond Harris (Vanderbilt University). C57Bl/6 Jak2V617F-MX1-Cre+ mice and C57Bl/6 Cd45.1Pep Boy mice were obtained from Jackson Laboratories (strain numbers: 037558 and 002014, respectively).<br><br>For the Tet2 studies: At 8-9 weeks of age, mice underwent bone marrow transplantation and then underwent kidney injury studies 6-8 weeks later (after sufficient bone marrow engraftment).<br>For the Jak2 studies: At 8-9 weeks of age, the heterozygous Jak2V617F mice were injected with 20 µg/g body weight of Polyinosinic–polycytidylic acid (PipC) three days a week for two weeks. Then, the mice were subjected to ischemia-reperfusion or unilateral ureteral obstruction immediately following this two-week period.
Wild animals	No wild animals were used in the study
Reporting on sex	Tet2 mouse experiments were conducted in male mice, while Jak2 mouse experiments were conducted in both male and female mice.
Field-collected samples	No field-collected samples were used in the study.
Ethics oversight	The Vanderbilt University Medical Centre animal care committee approved all animal study procedures.<br>Tet2 mice were initially bred at Queen's University (approved University Animal Care Committee protocol 2021-2128).

Note that full information on the approval of the study protocol must also be provided in the manuscript.
