# Supplementary figures and images for: Clonal hematopoiesis of indeterminate potential is associated with acute kidney injury
Source: Nat Med. 2024 Mar 7;30(3):810–7. doi: 10.1038/s41591-024-02854-6 (PMC10957477; doi:10.1038/s41591-024-02854-6)

Figure 3 B.

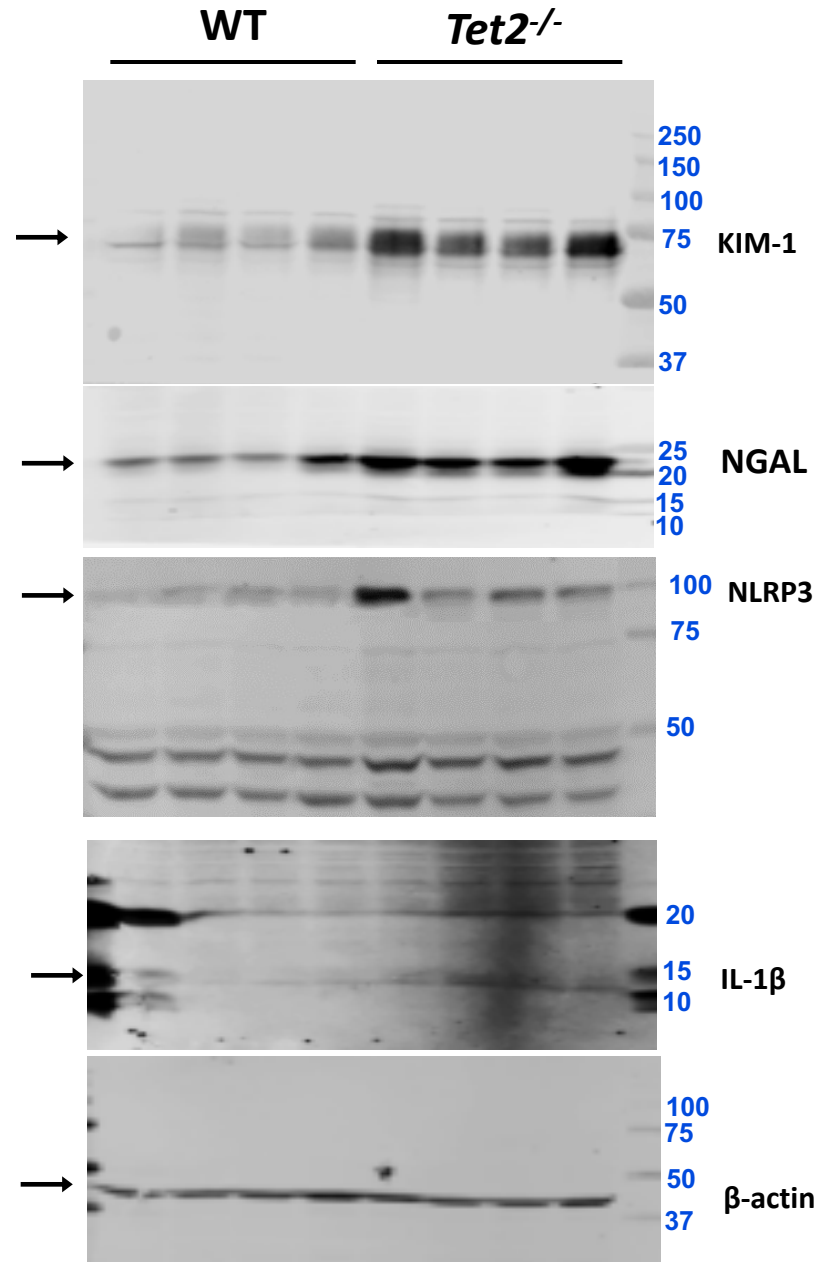

Supplement: Supplementary file 3 — Unprocessed immunoblots. [file 41591_2024_2854_MOESM3_ESM.pdf]

Figure 5 B.

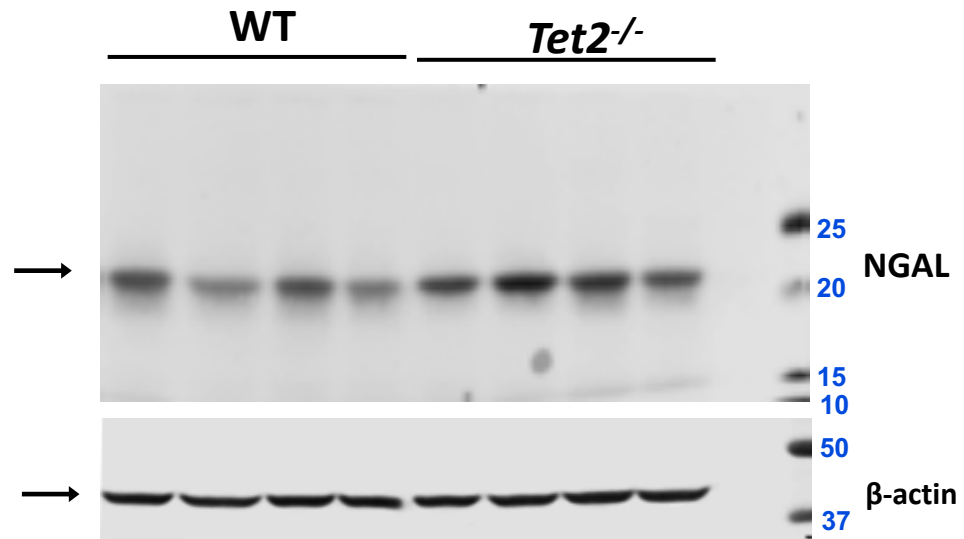

Supplement: Supplementary file 4 — Unprocessed immunoblots. [file 41591_2024_2854_MOESM4_ESM.pdf]

Figure 6 D.

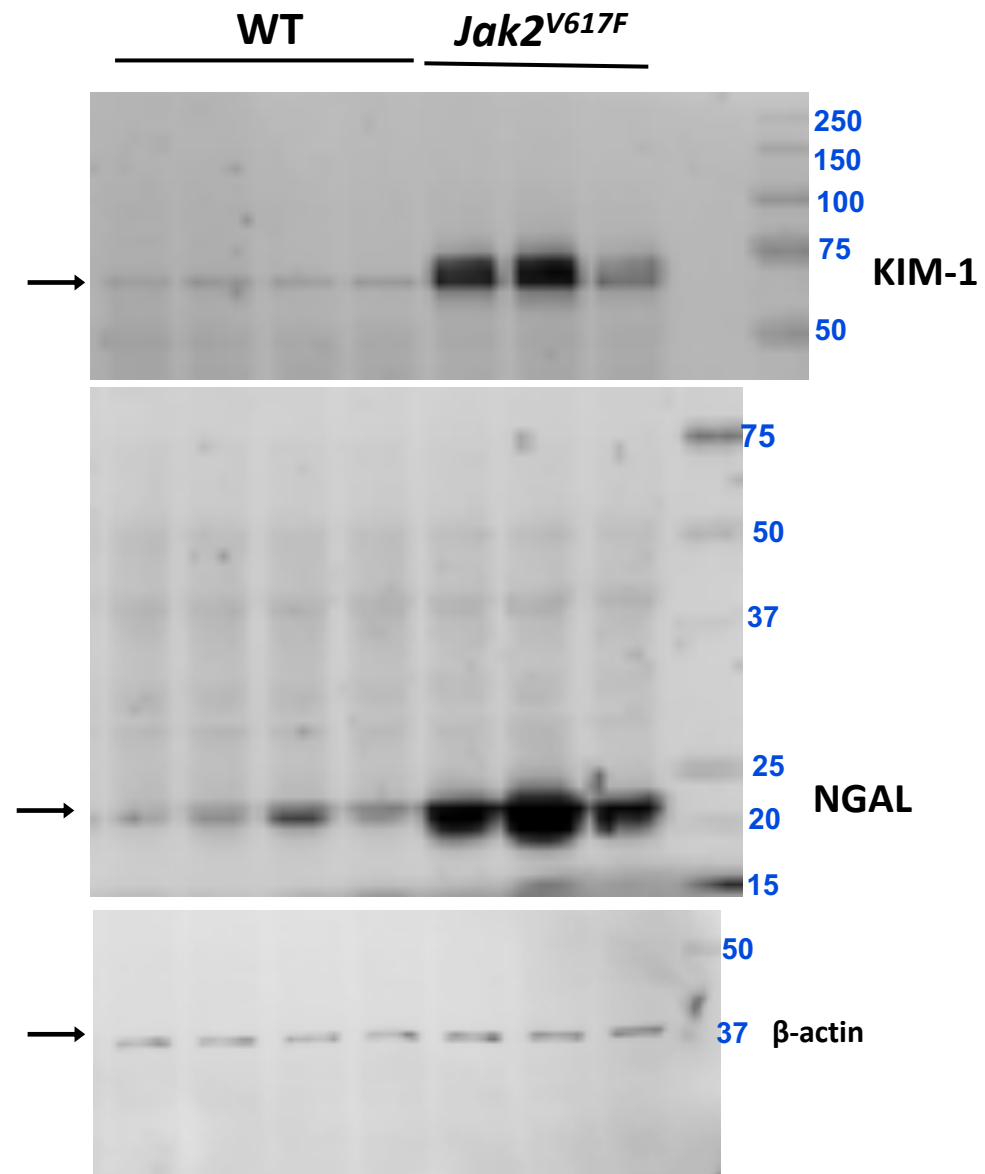

Figure 6 G.

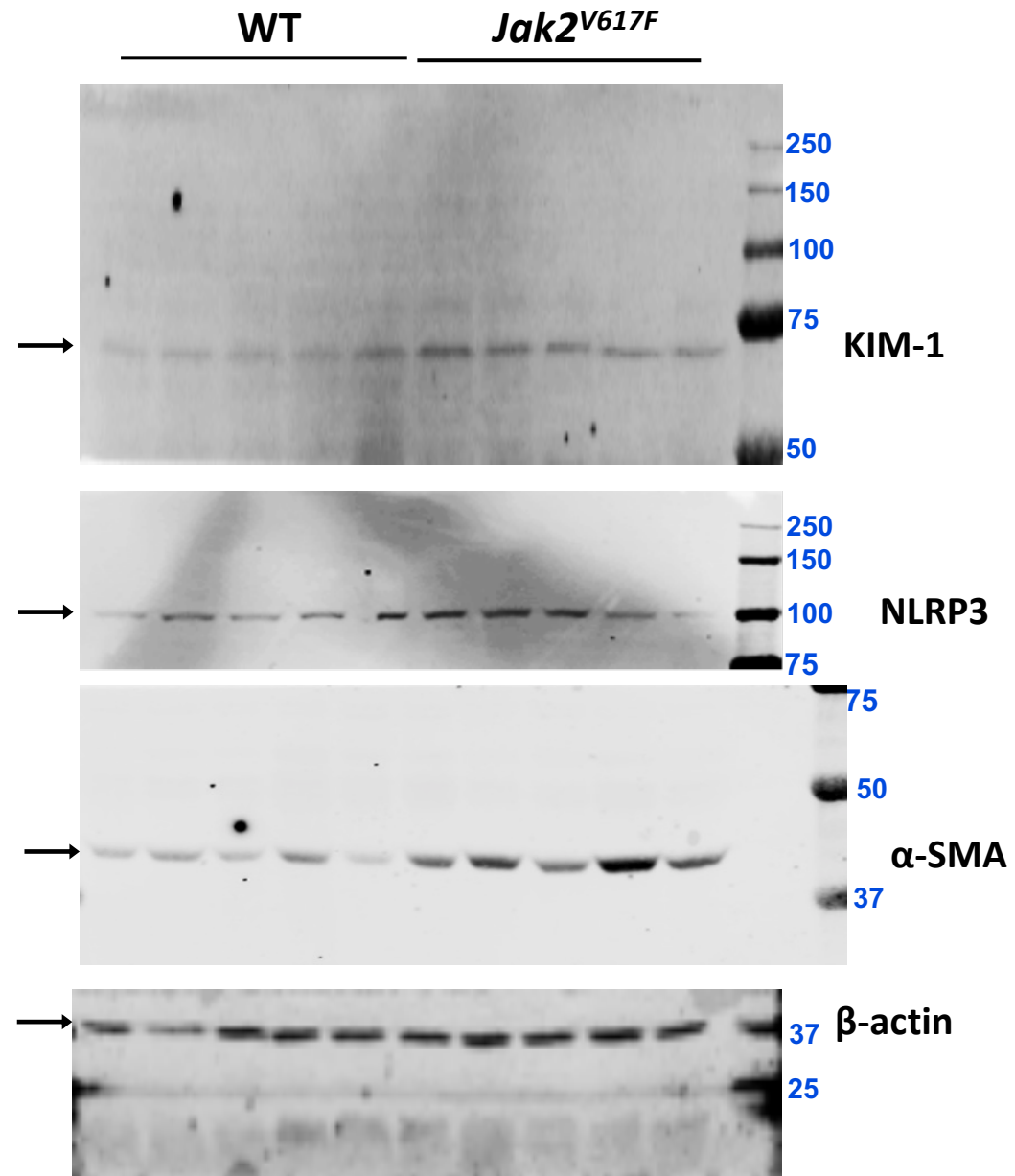

Figure 6 J.

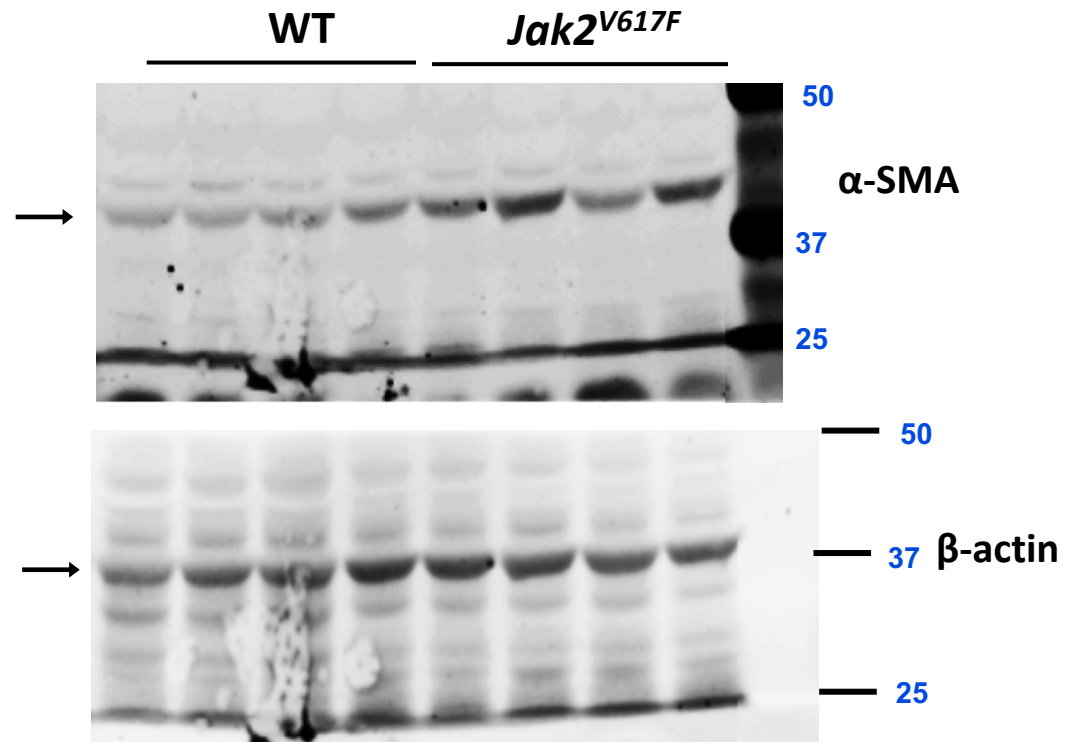

Supplement: Supplementary file 5 — Unprocessed immunoblots. [file 41591_2024_2854_MOESM5_ESM.pdf]
